# Insights into the Steps of Breast Cancer–Brain Metastases Development: Tumor Cell Interactions with the Blood–Brain Barrier

**DOI:** 10.3390/ijms23031900

**Published:** 2022-02-08

**Authors:** Fabienne Hamester, Christine Stürken, Ceren Saygi, Minyue Qi, Karen Legler, Christian Gorzelanny, José R. Robador, Barbara Schmalfeldt, Elena Laakmann, Volkmar Müller, Isabell Witzel, Leticia Oliveira-Ferrer

**Affiliations:** 1 Department of Gynecology, University Medical Center Hamburg-Eppendorf, 20251 Hamburg, Germany; k.legler@uke.de (K.L.); b.schmalfeldt@uke.de (B.S.); e.laakmann@uke.de (E.L.); v.mueller@uke.de (V.M.); i.witzel@uke.de (I.W.); ferrer@uke.de (L.O.-F.); 2 MSH Medical School Hamburg, Faculty of Medicine, Medical University, 20251 Hamburg, Germany; c.stuerken.ext@uke.de; 3 Department of Gynecology, Anatomy and Experimental Morphology, University Medical Center Hamburg-Eppendorf, 20251 Hamburg, Germany; 4 Bioinformatics Core, University Medical Center Hamburg-Eppendorf, 20251 Hamburg, Germany; c.saygi@uke.de (C.S.); m.qi@uke.de (M.Q.); 5 Department of Dermatology and Venerology, University Medical Center Hamburg-Eppendorf, 20251 Hamburg, Germany; c.gorzelanny@uke.de (C.G.); jose.robador@gmail.com (J.R.R.)

**Keywords:** breast cancer–brain metastasis, breast cancer molecular subtypes, blood–brain barrier, adhesion, invasion, gap junction assembly, angiogenesis

## Abstract

Brain metastases (BM) represent a growing problem for breast cancer (BC) patients. Recent studies have demonstrated a strong impact of the BC molecular subtype on the incidence of BM development. This study explores the interaction between BC cells of different molecular subtypes and the blood–brain barrier (BBB). We compared the ability of BC cells of different molecular subtypes to overcome several steps (adhesion to the brain endothelium, disruption of the BBB, and invasion through the endothelial layer) during cerebral metastases formation, in vitro as well as in vivo. Further, the impact of these cells on the BBB was deciphered at the molecular level by transcriptome analysis of the triple-negative (TNBC) cells themselves as well as of hBMECs after cocultivation with BC cell secretomes. Compared to luminal BC cells, TNBC cells have a greater ability to influence the BBB in vitro and consequently develop BM in vivo. The brain-seeking subline and parental TNBC cells behaved similarly in terms of adhesion, whereas the first showed a stronger impact on the brain endothelium integrity and increased invasive ability. The comparative transcriptome revealed potential brain-metastatic-specific key regulators involved in the aforementioned processes, e.g., the angiogenesis-related factors TNXIP and CXCL1. In addition, the transcriptomes of the two TNBC cell lines strongly differed in certain angiogenesis-associated factors and in several genes related to cell migration and invasion. Based on the present study, we hypothesize that the tumor cell’s ability to disrupt the BBB via angiogenesis activation, together with increased cellular motility, is required for BC cells to overcome the BBB and develop brain metastases.

## 1. Introduction

Breast cancer (BC) is the second leading cause of brain metastases (BM) after lung cancer. About 10–20% of all metastatic BC patients develop BM and the incidence continues to rise [1,2]. The frequency of BM has increased, particularly in recent years, as the treatment options for primary BC have developed and patients live longer. At the same time, the detection options for BM have improved, resulting in much more frequently diagnosed BM and an increasing incidence [3,4]. Unfortunately, patients who develop BM still have a poor prognosis and a short overall survival time. Despite treatment, the median life expectancy after diagnosis is only 2 to 25 months [5,6,7]. BM are often associated with neurological deficits, which include both cognitive and sensory dysfunction [8]. Affected patients have to accept an enormous loss in their quality of life [9]. The development of breast cancer–brain metastases (BCBM) is, in most cases, preceded by the existence of metastases in other organs such as the lung, liver, or bones [1]. In the metastatic setting, various therapy options are offered nowadays, including surgical removal of metastases, whole-brain radiotherapy, stereotactic radiation, and targeted therapies, but the chances of complete remission are often limited [5,10,11]. It is, therefore, imperative to better understand the molecular mechanisms of BM development and growth in order to predict BM and to improve the treatment for these patients. The subtype of the primary tumor is decisive for the prognosis and the treatment decision, thus representing a strong prognostic factor [12,13,14,15]. Depending on the expression or lack of certain receptors, tumors can be classified into one of the following three main groups: estrogen/progesterone-receptor-positive (ER+/PR+), human epidermal growth factor receptor 2 positive (HER2+), and triple-negative (TNBC), the latter showing the worst survival rate and, therefore, representing the most aggressive form [14]. Concerning the development of BM, previous studies have shown that patients of the TNBC and HER2+ subtype have a higher risk of developing BM, and several factors associated with BM in HER2-positive and TNBC have been identified [16,17,18,19]. BM development is a complex and multistage process. Tumors of various entities have to pass this process, which is also known as the metastatic cascade, to ultimately form metastases in another organ [20].

BM arises mainly through the hematogenous route, either directly from the primary tumor or from other organs in which metastases have already formed [21,22]. Once in the bloodstream system, tumor cells are arrested in the small capillaries of the blood vessels in order to pass the blood–brain barrier (BBB). Due to its special composition including several cell types, the BBB has unique properties that do not occur in other areas of the body. Every cellular component is essential to build a tight barrier that protects the central nervous system controlling the cell and molecular trafficking [23,24,25]. The neurovascular unit of the BBB is formed by endothelial cells, which line the blood vessels and form a continuous and impermeable layer characterized by the expression of tight-junction proteins and a surrounding basement membrane [26]. After the tumor cells have managed to penetrate the highly selective BBB, they might proliferate again and form metastases [25,27,28]. Only a few tumor cells can go through all of these steps and ultimately form metastases [29]. Thus, individual tumor cells might possess a certain genetic predisposition but also the ability to subsequently grow within the brain. Here, the interaction between tumor and brain cells is essential to successfully adapt to the brain environment.

This study aimed to uncover specific properties of BC cells that metastasize into the brain, especially those associated with the interaction between tumor cells and the blood–brain barrier. Thus, a better understanding of the precise mechanisms of BM might help to identify novel predictive factors and new therapeutic targets.

## 2. Results

### 2.1. Different Properties of Luminal and Triple-Negative Breast Cancer Cells during BBB Extravasation: Adhesion, Permeabilization Capacity, and Invasion

BC cell lines of different molecular subtypes and metastatic potential were analyzed regarding their ability to attach to, disrupt, and invade the brain endothelium. The estrogen-receptor-positive (ER+) cell line MCF-7 is known to have low invasive potential, whereas the TNBC cell line MDA-MB-231 is highly invasive. Properties of the brain seeking subline MDA-MB-231-BR, which represents a BC cell line that develops BM in vivo, were investigated, as well. Further, for the BBB model, we chose primary human brain endothelial cells (hBMEC) instead of a brain endothelial cell line, as primary cells have been described to preserve the native cell morphology and maintain many key features of the BBB in vitro [30,31,32].

**Figure 1:**
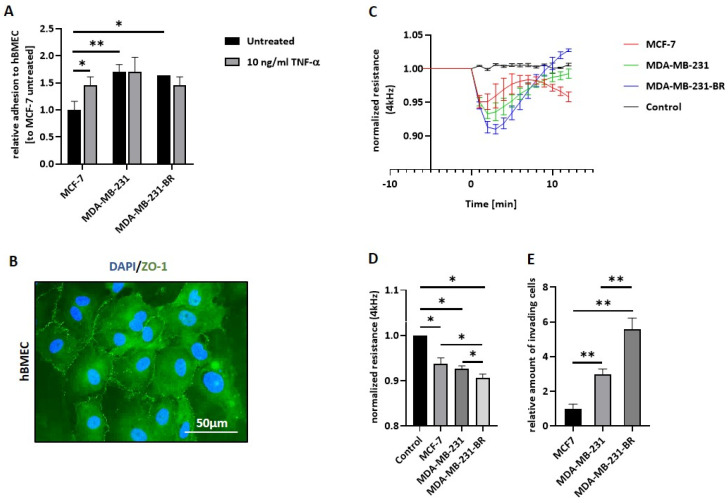
Properties of BC cell lines during brain metastatic cascade. (**A**) BC cell (MCF-7, MDA-MB-231, and MDA-MB-231-BR) adhesion to activated (+TNF-α, 10 ng/mL for 4 h) and not activated (-TNF-α, untreated) hBMECs was analyzed under static conditions. Relative amount (to MCF-7 untreated situation = 1) of adhesive cells is shown (representative experiment; *n* = 5); (**B**) immunofluorescence staining of ZO-1 (green) and nuclei (DAPI, blue) in hBMECs, magnification 60×; (**C**) the effect of different BC cells on BBB integrity are shown as normalized resistance values at 4 kHz (values were set at 1 before treatment (=0 min)) measured with the ECIS system over 20 min (representative experiment; *n* = 3); **(D**) bar graphs displaying relative resistance values under the influence of different tumor cells (*n* = 3); (**E**) invasion potential of MCF-7, MDA-MB-231, and MDA-MB-231-BR through hBMECs measured in a transwell assay (*n* = 3). Values are means ± s.d. * *p* < 0.05, ** *p* < 0.005.

Adhesion to the brain endothelium represents the first essential step during the brain metastatic cascade. Here, the adhesive potential of the aforementioned BC cell lines to an hBMECs monolayer was investigated under static conditions (Figure 1A). Both TNBC cell lines MDA-MB-231 and MDA-MB-231-BR adhered significantly more strongly to the brain endothelium than the low-invasive luminal MCF-7 (*p* = 0.004; *p* = 0.012). Contrary to our expectations, the brain metastatic subline MDA-MB-231-BR did not show a stronger adhesion ability to the brain endothelium compared to the parental cell line. Additionally, we analyzed the adhesion to hBMECs after activation with tumor necrosis factor-α (TNF-α). TNF-α is known to enhance the expression of several endothelial adhesion molecules, such as selectins. After 4 h of stimulation, there was no statistically significant difference in the number of adherent cells, neither for MDA-MB-231 nor for MDA-MB-231-BR, suggesting that these cells release their own endothelial cell-stimulating factors. In contrast, a significant increase in adhesion to the activated hBMECs was observed for MCF-7 cells (*p* = 0.024), suggesting a pronounced selectin-dependent adhesion for these cells.

In order to study the impact of the different BC cell lines on the integrity of the BBB, we used the electric cell-substrate impedance sensing (ECIS) system and the primary brain endothelial cells (hBMECs), which were seeded on gold electrodes in specialized chamber slides. This system allowed us to quantify the impact of breast cancer cells on the endothelial cell barrier in realtime and with high sensitivity [33]. Precisely, we analyzed the impedance at 4 kHz over 20 min, as this frequency has shown the highest sensitivity across endothelial cell monolayers [34]. A membranous ZO-1 expression, a tight-junction protein known to be essential for the generation of the BBB, on the hBMEC monolayer was confirmed previous to the ECIS experiments via immunofluorescence staining (Figure 1B). The ability of the aforementioned BC cell lines to open the tight BBB-like endothelial cell monolayer is shown in Figure 1C. Resistance values before the addition of tumor cells (time point 0 min) were set at 1 and subsequently, MCF-7, MDA-MB-231, and MDA-MB-231-BR cells were seeded on the EC monolayer at an endothelial–tumor cell ratio of 1:1. The graphic in Figure 1C shows a clear and rapid decrease, within the first 10 min, in the barrier resistance for all BC cell lines, compared to the control (medium) after BC cell addition. For all three cell lines, a short opening of the BBB with the strongest effect for the MDA-MB-231-BR cells could be observed. Figure 1D shows the summary of three independent experiments. Here, all investigated BC cell lines were able to significantly decrease the resistance (control vs. MCF-7 *p* = 0.001; control vs. MDA-MB-231 *p* < 0.005; and control vs. MDA-MB-231-BR *p* = 0.003) and, therefore, to impact the integrity of the BBB. Interestingly, the impact on the endothelial cell monolayer permeability caused by the brain-seeking cell line MDA-MB-231-BR was significantly stronger than with the parental cell line MDA-MB-231 or the low invasive MCF-7 cells, whereas no significant difference could be observed between MCF-7 and MDA-MB-231 cells (MCF-7 vs. MDA-MB-231 *p* = 0.307; MCF-7 vs. MDA-MB-231-BR *p* = 0.029; and MDA-MB-231 vs. MDA-MB-231-BR *p* = 0.027).

Moreover, the invasive potential of the different BC cells through a brain endothelial cell layer was analyzed using a transwell system. The hBMECs were seeded on a porous membrane and grown to confluence and tumor cells, which were labeled with a green fluorescence dye, seeded on top, and allowed to invade through the endothelial monolayer for 48 h. The results are shown in Figure 1. Here, the brain metastatic cell line showed the highest invasion potential, with a significant difference compared to MDA-MB-231 (2-fold, *p* < 0.005) as well as to MCF-7 (3-fold, *p* < 0.005). In addition, MDA-MB-231 can significantly more strongly invade the brain endothelium compared to the low-invasive MCF-7 cells (*p* < 0.005).

### 2.2. Different Invasive Behavior of Luminal and Triple-Negative Breast Cancer Cells after Intracardiac Injection in Mice

**Figure 2:**
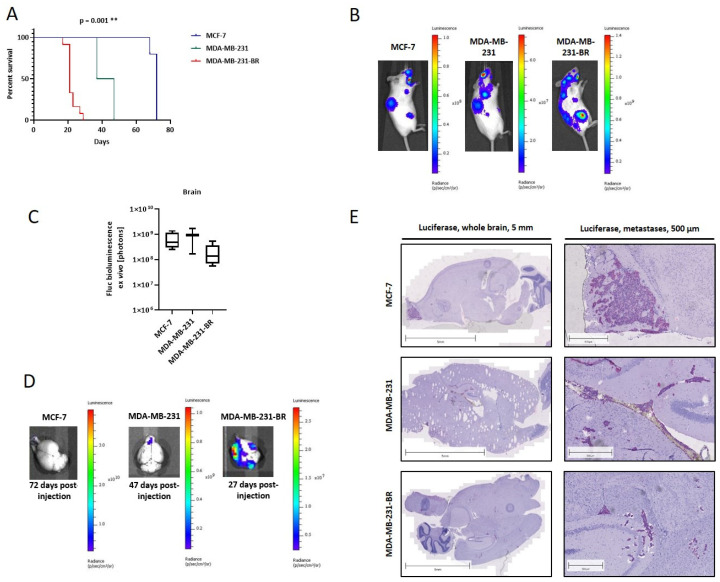
BM development in an intracardiac mouse model depending on different BC cell lines. (**A**) Kaplan–Meier plot of mouse survival, intracardiac injected with 1 × 10^6^ cells of each BC cell line (MCF-7: *n* = 5, MDA-MB-231: *n* = 2, MDA-MB-231-BR: *n* = 12); (**B**) representative BLI pictures of whole mice of each group 21 days after injection; (**C**) ex vivo BLI signal quantification from brains of all three test groups (MCF-7, MDA-MB-231, and MDA-MB-231-BR); (**D**) representative pictures of BLI-measured brains at the final time point of each group; (**E**) sagittal sections of mouse brains immunohistochemically stained on luciferase. From each test group, one representative picture of the whole brain and detailed metastases staining are shown. Corresponding scales are indicated above the pictures. Values are means ± s.d. ** *p* < 0.005.

The metastatic potential of the three aforementioned BC cell lines was subsequently analyzed in an in vivo model based on the intracardiac injection of tumor cells, which has been described as successfully leading to brain metastasis formation [35]. Here, 1 × 10^6^ cells from each BC cell line (MCF-7, MDA-MB-231, and MDA-MB-231-BR) were intracardially injected into the left heart ventricle of 6-week-old female SCID mice (*n* = 15 per group). Several mice were removed from the experiment either on the injection day or shortly after that, showing evident poor condition or strong bioluminescence signals in the lungs. Unfortunately, the resulting groups were markedly reduced and heterogeneous, namely *n* = 5, *n* = 2, and *n* = 12 mice injected with MCF-7, MDA-MB-231, and MDA-MB-231-BR, respectively. Despite this limitation in the experimental setting, we observed a clear trend regarding the metastatic potential among the three cell lines. Here, MDA-MB-231-BR showed the most aggressive behavior with an average survival time of the injected mice of 21 days, followed by the parental cell line (42 days) and finally the luminal MCF-7 cell line (72 days) (Figure 2A).

Remarkably, the experimental endpoint for the MCF-7- and MDA-MB-231-injected mice was set due to high weight loss (>10% of starting weight) and a resulting poor general condition, whereas in 92% of the MDA-MB-231-BR cell-injected mice, paralysis of the hindquarters led to the termination of the experiment. The weekly bioluminescence measurement supported this finding, as shown exemplarily in Figure 2B for one mouse corresponding to each group at day 21 after tumor cell injection, displaying the strongest bioluminescence signals, as well as the highest extent of tumor spread in the brain-seeking cell line mouse group. The quantification of circulating tumor cells (CTCs) in the blood and disseminated tumor cells (DTCs) in the bone marrow at the endpoint showed the same trend, although for the CTCs, differences were not statistically significant (data not shown). The ex vivo bioluminescence at the endpoint showed significant differences in the gastrointestinal tract (GIT) and the kidneys. While for the GIT, a significant difference could be found only between the MDA-MB-231 and the MDA-MB-231-BR groups, the MCF-7 group showed a significantly higher metastasis burden of the kidneys compared to the TNBC groups (data not shown). BM were observed not only in the MDA-MB-231-BR group as expected but also in the other two groups. Figure 2D shows representative BLI images at the endpoint from each group, as well as the corresponding quantification (Figure 2C) including all analyzed mice. Here, no significant differences between the groups could be detected, probably due to the different experimental endpoints for each group. However, it has to be taken into consideration that signal intensities around 1 × 10^8^ were reached in the MDA-MB-231-BR group after only 21 days, whereas 42 and 72 days on average are required in the corresponding parental cell line MDA-MB-231 and MCF-7 test group, respectively. The presence of brain metastatic lesions was confirmed histologically using luciferase staining as described in Materials and Methods. BM was identified in all 12 mice injected with MDA-MB-231-BR cells as well as in the group injected with MDA-MB-231. However, despite the strong BLI signals, only in three of the five mice injected with MCF-7 cells could BM be histologically proven. In the MCF-7 mouse group, large macrometastases in the olfactory bulb were found, whereas smaller and micrometastatic lesions were detected all across the cerebrum and to some extent in the cerebellum in the MDA-MB-231 and MDA-MB-231-BR injected mice, respectively (Figure 2E).

### 2.3. Influence of the Breast Cancer Secretome on Brain Endothelium

**Figure 3:**
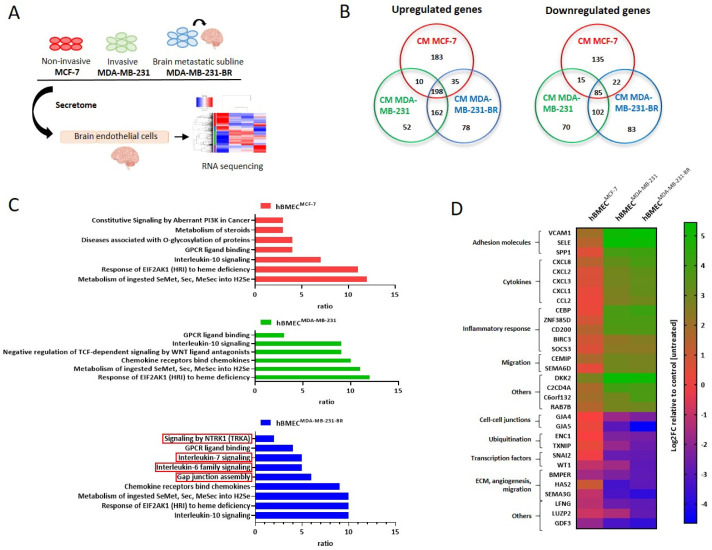
Transcriptome analysis of hBMECs after treatment with BC secretomes. (**A**) Schematic representation of the experimental design: hBMECs were treated with CM of BC cell lines MCF-7, MDA-MB-231, and their corresponding brain metastatic subline (MDA-MB-231-BR), and RNA sequencing was subsequently performed; (**B**) Venn diagrams showing RNA sequencing results (*n* = 4). A total of 58.611 genes were analyzed from each data set. The number of up- and downregulated and overlapping genes compared between different groups of treatment (hBMEC^Ctrl^, hBMEC^MCF-7^, hBMEC^MDA-MB-231^, and hBMEC^MDA-MB-231-BR^) are displayed; (**C**) reactome pathway analysis represents pathways enriched due to different treatments; (**D**) heatmap displaying log2FC values of the strongest differential gene expression by the influence of TNBC secretomes relative to control (untreated).

To obtain insights into the molecular mechanisms of how tumor cells impact the BBB integrity, the effect of the conditioned media (CM) from the three aforementioned BC cell lines on brain endothelial cells was analyzed by RNAseq. Here, hBMECs were incubated for 4 h with CM from MCF-7, MDA-MB-231, and MDA-MB-231-BR as well as with medium as a control. For each of the 4 treatments (hBMEC^Ctrl^, hBMEC^MCF-7^, hBMECM^DA-MB-231^, and hBMEC^MDA-MB-231-BR^) four replicates were prepared, followed by RNA isolation and RNAseq analysis, as schematically represented in Figure 3A.

The differentially expressed endothelial genes of all three groups are summarized in the Venn diagrams (Figure 3B). Compared to the control, which represents hBMECs treated with the medium, all three cell lines strongly influenced the brain endothelial transcriptome. However, we found remarkable differences in the kinds of genes and pathways deregulated by the different subtypes. Deregulated genes were mapped to cellular pathways from the Reactome database and displayed in Figure 3C. While several pathways, such as interleukin-10 signaling, were commonly deregulated in ECs treated with all three secretomes, others like chemokine receptors bind chemokines were only deregulated in endothelial cells treated with CM from TNBC cell lines. Remarkably, four pathways were specifically deregulated by the brain-seeking cell line, namely signaling by NTRK1, interleukin-7 signaling, interleukin-6 family signaling, and gap junction assembly. Regarding single deregulated genes, both TNBC cell lines commonly upregulated 162 and downregulated 102 genes (Figure 3B) and in addition, several genes were deregulated by all subtypes but significantly more strongly by the TNBC compared to the ER+ cells. Among them, we identified adhesion molecules, signaling molecules including cytokines, transcription factors, genes involved in metabolic processes and nervous system development, as well as transmembrane transporters (Table S1A,B). From these TNBC-specifically deregulated genes, the 31 strongest regulated ones are shown as a heatmap in Figure 3D. Remarkably, the strongest deregulated factors were two cell adhesion molecules (CAMs), namely E-selectin (*SELE*) and vascular cell adhesion protein 1 (*VCAM1*) with log2FC values of 5.4 and 5.2, respectively. These data could be validated at the mRNA level and the protein level via qRT-PCR and flow cytometry analyses (Figure S1), underlying the essential role of these two endothelial cell adhesion molecules for the attachment of TNBC cells to the brain endothelium. Further upregulated genes included cyto- and chemokines (e.g., *CXCL1/2/3/8* and *CCL2*), genes associated with the inflammatory response (e.g., *CEBP* and *SOCS3*), and genes related to cell migration (*CEMIP* and *SEMA6D*). Remarkably, among the downregulated genes, we found cell–cell junction molecules such as *GJA4* and *GJA5*.

### 2.4. Brain Metastatic Specific Features of Triple-Negative Breast Cancer Cells

**Figure 4:**
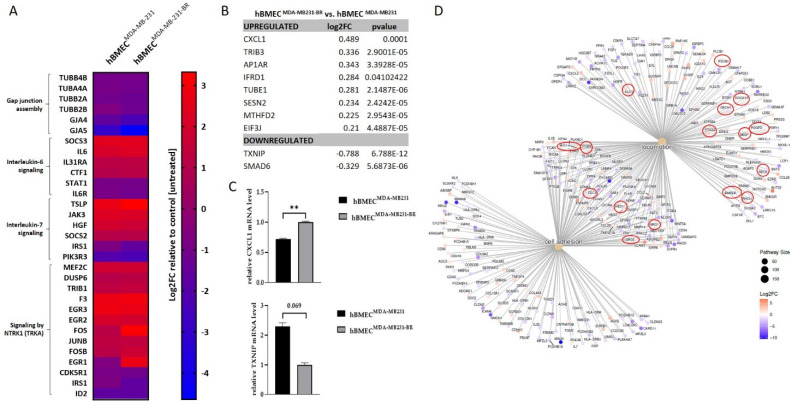
Brain metastatic-specific effects of BC cells on the brain endothelium. (**A**) Heatmap displaying log2FC values corresponding to all genes included in signaling by NTRK1, interleukin-7 signaling, interleukin-6 family signaling, and gap junction assembly pathways for hBMEC^MDA-MB-231-BR^ as well as hBMEC^MDA-MB-231^, both vs. hBMEC^Ctrl^; (**B**) list of endothelial genes significantly deregulated by MDA-MB-231-BR in comparison to the parental cell line MDA-MB-231; (**C**) relative brain endothelial expression of *CXCL1* and *TXNIP* after 4 h of treatment with CM of MDA-MB-231 (hBMEC^MDA-MB-231^) and CM of MDA-MB-231-BR (hBMEC^MDA-MB-231-BR^); (**D**) gene concept network displaying significantly differentially expressed genes of MDA-MB-231-BR cells compared with MDA-MB-231 in enriched GO-terms cell adhesion and locomotion. Genes of interest are marked up. Values are normalized to corresponding *GAPDH* expression (*n* = 3). Values are means ± s.d. ** *p* < 0.005.

As mentioned before, four pathways were significantly deregulated in ECs after treatment with MDA-MB-231-BR secretome compared with control-treated ECs. The heatmap in Figure 4A shows log2FC values corresponding to all genes included in the aforementioned pathways for hBMEC^MDA-MB-231-BR^ as well as hBMEC^MDA-MB-231^, both vs. control EC (hBMEC^Ctrl^) Here, we observed that most factors were significantly deregulated by both TNBC cell lines, although always to a higher extent by the brain-seeking cell line. Two gap junction proteins (*GJA5* and *GJA4*) as well as several tubulin chain proteins (*TUBB4B*, *TUBA4A*, *TUBB2A*, and *TUBB2B*) were significantly downregulated in ECs after treatment with the TNBC cells. Additionally, a significant upregulation of several factors involved in interleukin-6 (*SOCS3*, *IL6*, *IL31RA*, and *CTF1*) and interleukin-7 (*JAK3*, *SOCS2*, *TSLP*, and *HGF*) signaling was noted, whereas four genes related to these pathways were downregulated (*STAT1*, *IL6R*, *PIK3R3*, and *IRS1*). Moreover, the signaling by the NTRK1 pathway was enriched, including 10 upregulated genes (*MEF2C*, *DUSP6*, *TRIB1*, *EGR1*, *F3*, *JUNB*, *FOS*, *EGR3*, *FOSB*, and *EGR2*) and two downregulated factors (*CDK5R1* and *ID2*).

Surprisingly, when comparing the effect of the brain-seeking subline with the parental cell line, we found few significantly deregulated genes. Merely 10 endothelial genes, which were significantly more strongly deregulated by MDA-MB-231-BR than by the parental cell line MDA-MB-231, could be identified (Figure 4B). Here, although the RNA levels were significantly different, the extent of the deregulation (log2FC) was very low. The gene most upregulated in the brain endothelial cells by the influence of MDA-MB-231-BR was *CXCL1* (log2FC hBMEC^MDA-MB-231-BR^ vs. hBMEC^MDA-MB-231^ = 0.49) and the most downregulated gene was *TXNIP* (log2FC hBMEC^MDA-MB-231-BR^ vs. hBMEC^MDA-MB-231^ = −0.79). As both genes have been described to play a role during angiogenesis and might be important during the brain metastatic process, a validation at the mRNA level via qRT-PCR (Figure 4C) was performed. Here, MDA-MB-231-BR CM significantly upregulated *CXCL1* (*p* = 0.0001) and almost significantly downregulated the *TXNIP* gene level (*p* = 6.788 × 10^−12^) in hBMECs in comparison to MDA-MB-231 CM.

Since the paracrine effect of the parental and the corresponding brain-seeking cell line on the brain endothelium was rather similar, we took a deeper look at the molecular characteristics of both cell lines themselves using RNAseq analysis. According to the over-representation analysis, significantly differentially expressed genes in the brain-seeking cell line compared with the parental cell line are significantly enriched, primarily in GO-terms extracellular matrix organization (enrichment ratio: 2.53, FDR: 5.9 × 10^−11^), cell junction organization (enrichment ratio: 2.08, FDR: 3.2 × 10^−11^), cell adhesion (enrichment ratio: 1.9, FDR: 8.1 × 10^−15^), cell motility (enrichment ratio: 1.73, FDR: 1.0 × 10^−12^), and locomotion (enrichment ratio: 1.69, FDR: 6.5 × 10^−31^). Among all genes included in these categories (Figure 4D), we would like to highlight several interesting candidates involved in angiogenesis (*PTGS2*, *SLIT2*, *JAG1*, *RECK*, and *BMPER*), cytoskeleton reorganization (*MISP*, *FSCN1*, and *DLC1*), and/or tumor cell migration and motility (*PTGS2*, *JAG1*, *PDGFD*, *MISP*, *HES1*, *NRG1*, *ITGB3*, *DOCK10*, *GPC6*, *FSCN1*, *DACH1*, *RHOU*, *RECK*, and *DLC1*).

## 3. Discussion

Breast cancer brain metastases (BCBM) have become a critical issue due to their increasing incidence and the lack of effective treatment. To develop successful therapy options for BCBM patients, but also in order to predict a predisposition of primary BC patients to develop BM, we must improve our knowledge of the molecular characteristics required for tumor cells to interact and successfully pass the blood–brain barrier. In this context, it is well known that the histological subtype of BC patients is strongly associated with the development of BM. The risk of BM formation in patients of luminal subtype is lower than in triple-negative and HER2-positive patients, with incidence rates of 30–40% for the last two subtypes. Here, the present study aimed to identify key molecular players specifically involved in tumor cell adhesion to the brain endothelium, tumor cell disruption of the BBB, and tumor cell migration through the BBB. We have, therefore, compared the functional and molecular characteristics of one luminal BC cell line (MCF-7) and two well-established TNBC cell lines, namely MDA-MB-231 and its brain-seeking subline MDA-MB-231-BR, regarding the aforementioned three key metastatic steps.

Once tumor cells have entered the lymphatic or vascular system and have been able to survive the shear forces of blood flow and evade the immune system, they must extravasate at distant sites in order to successfully metastasize. Extravasation is, therefore, a key step during metastasis and comprises a cascade of events including the initial tumor cell adhesion to the endothelial layer and the subsequent tumor cell transendothelial migration. To mimic this specific step of the metastatic cascade, we have performed an in vivo model with all three aforementioned breast cancer cell lines, in which tumor cells were delivered directly into the arterial blood supply of SCID mice after intracardiac injection. Despite the limitations of the experiment, due to a reduced number of animals in one of the groups, we observed a higher metastatic potential of the triple-negative cell lines in comparison with the luminal one, particularly in the brain-seeking cell line group, as previously described [36]. Remarkably, the histological examination of the mouse brains ex vivo highlighted this finding and showed a significantly increased amount of brain lesions in mice injected with TNBC cells than in those with the luminal cell line MCF-7. Moreover, the localization of the metastases within the brain strongly differed between both molecular subtypes, in line with published data corresponding to BCBM patients [37,38]. The results of the intracardiac model highlight the fact that TNBC cells might display certain molecular characteristics, which provide an advantage during extravasation through the BBB, thereby leading to increased BM formation. In order to take a deeper look into the extravasation process, we subsequently studied separately three different extravasation phases in vitro, namely tumor cell adhesion, BBB disruption, and transendothelial tumor cell migration. The brain endothelium exhibits special features and strongly differs from endothelial cells of peripheral capillaries. The presence of tight junctions (TJ) without fenestrations especially leads to a relative lack of vesicular transport and a reduced diffusion rate. In our study, we have therefore performed all functional analyses with primary human brain microvascular endothelial cells, which have been described to adequately mimic the BBB properties [32,39]. As expected, both TNBC cell lines showed a significantly increased adhesion to brain endothelial cells in comparison with the luminal one. Interestingly, once endothelial cells were pretreated with TNFα, all cell lines exhibited comparable adhesion rates. Proinflammatory cytokines such as TNFα are known to modulate the surface levels of cell adhesion molecules; precisely for primary hBMECs, an increased expression of ICAM-1/2, VCAM-1, E-selectin, and ALCAM has been described after EC stimulation with TNFα [40]. Thus, we assume that in contrast to the luminal cell line MCF-7, TNBC cells can activate the endothelium through endogenous expression and secretion of proinflammatory factors, thereby leading to an efficient tumor cell adhesion, even in the absence of exogenous TNFα. Indeed, by RNAseq and FACS, we could confirm a strong upregulation of *SELE* and *VCAM1* in hBMECs after treatment with the conditioned medium (secretome) of both TNBC cell lines when compared with the stimulation using the MCF-7 cells’ secretome. Additionally, several cytokines and chemokines were upregulated in the brain endothelial cells exclusively in response to TNBC´s secretomes. Indeed, *CXCL8* and *CCL2* have been previously described as having increased primary HBMECs in response to tumor necrosis factor and/or interferon γ [41,42]. Whether these EC-secreted chemokines exert their function on tumor cells, immune cells, or on EC themselves is not known yet. In this context, it has been reported that CXCL8 triggers Akt/protein kinase B activation in brain endothelial cells, followed by a redistribution of tight junction structures and a destabilization of the BBB [43]. In line with this data, we observed a significantly reduced expression of two gap junction proteins, *GJA4* and *GJA5* in ECs treated with the secretome of TNBC cells in comparison with those stimulated with the CM of the luminal cell line. Gap junctions (GJ) belong beside the tight junctions and adherens junctions of the BBB junction complex. GJs are channel structures formed by members of the connexin family (Cx) that allow the transport of ions and small molecules between adjacent cells. Precisely, Cx37 (*GJA4*) and Cx40 (*GJA5*) are specifically expressed in brain endothelial cells. In this context, Nagasawa et al. showed a colocalization of Cx40 and Cx43 with the tight-junction molecules occluding, claudin-5, and ZO-1 in BBB endothelial cells, and further demonstrated that the gap junction’s inhibition disrupts the barrier function of tight junctions, leading to high transendothelial electrical resistance values and increased paracellular flux [44].

When comparing the adhesive properties of the two TNBC cell lines with each other in functional assays, no significant difference could be observed. These findings suggest that the increased brain-metastatic potential of the brain-seeking subline is not based on more advantageous adhesive properties, but rather on the capacity of these cells to effectively disrupt the endothelium and eventually migrate through this layer. Using an electric cell-substrate impedance sensing (ECIS) system, we could corroborate this assumption and show that, although all investigated BC cell lines significantly reduced the resistance of the endothelial layer, and therefore impacted the integrity of the BBB, compared to control conditions, particularly the brain-seeking subline achieved a significantly stronger barrier disruption. Here, comparative transcriptome analyses from brain endothelial cells treated with secretomes of both TNBC cell lines should help us to identify cellular structures affected specifically by paracrine stimulation with either the parental or the brain-seeking cells. Indeed, we identified four pathways that were specifically deregulated by the brain-seeking cell line in comparison to the control, namely signaling by NTRK1, interleukin-7 signaling, interleukin-6 family signaling, and gap junction assembly. However, a deeper look into the pathway-related factors revealed that most of them were not only significantly deregulated by the brain-seeking cell line but also by the parental TNBC cell line, although always to a lesser extent by the latter. To our surprise, only two endothelial genes were significantly deregulated in response to the brain-seeking cell line when compared with the parental cells, namely *CXCL1* and *TXNIP*. Here, one possible reason might be the similarity in the secretome composition of the two cells lines, as previously reported by Blache et al., who showed that the secretome of the brain-seeking BC subline is the least different from the parental cell line compared to other metastatic sublines (bone and lung) [45]. In addition, the duration of the secretomes’ treatment might also be decisive. A 4 hour treatment, as in the present study, might be too long to detect the deregulation of certain factors. In this context, the decrease of the BBB integrity measured by ECIS was observed within 10 min after adding the tumor cells and consequently, genes responsible for the BBB opening could be again expressed at normal levels after 4 h. Additionally, a redistribution of tight junction proteins, including occluding, claudin-5, and ZO-1, takes place during this process, which increases the permeability of the BBB but is not measurable at the transcription level.

Furthermore, we assume that after 4 hours of stimulation with the tumor cells´ secretome, we mainly detect deregulation of immediate-early genes, meaning a protein-independent induction, as previously described by Tullai et al. [46]. This assumption would indeed explain the small difference in the transcriptome of the brain endothelial cells. Further, one should consider that newly identified extracellular vesicles (EVs) could additionally impact the transcriptome of the stimulated brain endothelial cells. These tumor-derived EVs are important players in tumor progression and metastasis. EVs contain bioalogical molecules, such as nucleic acids (DNA, mRNA, microRNA, and other noncoding RNAs), proteins (receptors, transcription factors, enzymes, and extracellular matrix proteins), and lipids that can influence target cells such as brain endothelial cells [47,48]. Several studies already described the composition of the EVs for both the parental cell line MDA-MB-231 [49,50] and the brain-seeking subline MDA-MB-231-BR [51] to demonstrate a possible EV-dependent brain-metastatic-specific effect. Rodrigues et al. reveal only twenty proteins to be differentially expressed in brain-seeking breast cancer cell EVs (MDA-MB-231-BR) compared to the corresponding parental cell line EVs (MDA-MB-231). These include the cell migration-inducing and hyaluronan-binding protein CEMIP, as already described in this study to be upregulated under the influence of TNBCs´ secretome in brain endothelial cells, therefore suggesting a specific association with BCBM potential. Nishida-Aoki et al. recently demonstrated distinct glycosylation profiles in the brain-metastatic subline BMD2a in comparison to its parental human breast cancer cell line, MDA-MB-231-luc-D3H2LN by lectin blot [52]. The same group further showed that the aforementioned brain metastatic cancer cells release microRNA-181c-containing extracellular vesicles capable of destructing the blood–brain barrier [53].

As already mentioned, brain endothelial cells treated with the secretome of the brain-seeking cell line showed higher levels of *CXCL1* and lower levels of *TXNIP* in comparison to those stimulated with the secretome of the native cell line. The chemokine ligand 1 has been described elsewhere to reduce endothelial cell migration and proliferation by using neutralization antibodies against it [54]. In contrast, the gene silencing of *TXNIP*, a thioredoxin-interacting protein, stimulates endothelial migration, and vasculature network formation in human microvascular endothelial cells [55,56]. Thus, the literature attributes CXCL1 a proangiogenic role and TXNIP an antiangiogenic role. Taking the role of these two factors into account in the context of our RNAseq analysis, we can conclude that the brain metastatic subline stimulates the brain endothelium by activating angiogenesis, which is crucial for further tumor cell colonization in the brain.

In this context, the transcriptome analysis of both TNBC cell lines revealed several candidates that might be crucial for brain endothelium activation. Among them, *PTGS2* (*COS-2*) has been widely described as a key angiogenesis mediator [57] and involved in BBB disruption in the context of ischemic stroke [58]. Additionally, Slit2 induces tumor angiogenesis via Slit–Robo signaling and Slit2 overexpression in the mouse brain has been reported to increase blood vessel density and permeability [59,60]. Moreover, the bone morphogenetic protein modulator BMPER is highly expressed in malignant tumors and its loss has been shown to impair, among other cellular functions, tumor cell-induced endothelial cell sprout [61].

Platelet-derived growth factor D (PDGF-D) is a newly identified member of the platelet-derived growth factor (PDGF) family that binds exclusively to the PDGFRβ receptor. By binding to PDGFRβ on stromal cells, e.g., endothelial cells, PDGF-D promotes proliferation and angiogenesis. An autocrine effect has also been described on tumor cells, including TNBC cells, where PDFG-D stimulates cancer cell invasion [62]. Additional genes that might be responsible for the strong invasive and migratory ability of the brain-seeking subline could be identified in the comparative transcriptome analysis. Among them, mitotic spindle positioning factor (*MISP*) has been described to provide a link between the cell cortex and microtubule cytoskeleton, thereby affecting cell migration [63]. *HES1* (hairy and enhancer of split homolog-1), a transcriptional repressor involved in cell differentiation and proliferation as well as in cancer development, is overexpressed in breast cancer and especially in TNBC. Here, *HES1* downregulation in MDA-MB-231 cells led to decreased cell proliferation and invasion [64]. We also found a significantly higher expression of neuregulin 1 (*NRG1*) in MDA-MB-231-BR cells in comparison with the parental cell line. In this context, Cabrera et al. showed that tumor-secreted NRG1 stimulates macrophages to express JAG1 (jagged canonical Notch ligand 1), which in turn might activate Notch-associated signaling on endothelial cells, thereby promoting angiogenesis as well as on tumor cells leading to increased transendothelial migration and invasion [65]. Interestingly, we have also identified *JAG1* as one of the brain-metastatic-specific genes in our analysis, assuming that BC cell-secreted JAG1 might also act paracrinally as well as autocrinally on ECs and tumor cells, respectively. Integrin β3 might also play an important role in transendothelial cell migrations, as *ITGB3* downregulation has been demonstrated to inhibit cellular migration in TNBC cells [66]. Moreover, further significantly upregulated genes such as *DOCK10, GPC6*, and *FSCN1* have been related to enhanced tumor cell migration and/or invasion [67,68,69,70]. In contrast, several factors assigned to the suppression of tumor cell migration and/or invasion as well as angiogenesis, such as *DLC1*, *RHOU*, *DACH1*, and *RECK*, were significantly downregulated in the brain-seeking cell line in comparison with the native one [71,72,73,74,75,76].

Taken together, the present findings suggest that the tumor cells’ ability to activate the brain endothelium and, in turn, disrupt the BBB, together with a promigratory phenotype are required for BC cells to successfully develop brain metastasis. In this context, based on transcriptome analysis from tumor and brain endothelial cells, we have identified several molecular players that might play a key role in the aforementioned processes. To validate their functional relevance, further functional analyses are required.

## 4. Materials and Methods

### 4.1. Cell Lines

The human BC cell lines MDA-MB-231 and MDA-MB-231-BR (brain-seeking subline) were a kind gift from Professor Harriet Wikman–Kocher (Institute of Tumor Biology, University Medical Center Hamburg-Eppendorf, Hamburg, Germany). The MCF-7 cells were purchased from ATCC (American Type Culture Collection, Wesel, Germany). All were cultured in Dulbecco´s Modified Eagle Medium (DMEM, Thermo Fisher Scientific, Waltham, MA, USA) supplemented with 10% (*v*/*v*) fetal calf serum (FCS, Thermo Fisher Scientific, Waltham, MA, USA). Cell lines were recently authenticated at the DSMZ (German Collection of Microorganisms and Cell Cultures GmbH, Brunswick, Germany). Primary brain endothelial cells (hBMEC) were purchased from Cell Systems (#ACBRI376, Kirkland, WA, USA) and cultured with the corresponding Complete Classic Medium (#4Z0-500-R, Cell Systems, Kirkland, WA, USA) in Attachment Factor (#4Z0-201, Cell Systems, Kirkland, WA, USA) coated flasks. All cell lines were cultured under standard conditions in a water-saturated atmosphere containing 5% CO_2_ at 37 °C.

### 4.2. Conditioned Media Preparation

For conditioned media (CM) preparation, cell lines were grown first to 80% confluence, washed twice with Dulbecco’s Phosphate-Buffered Saline (PBS, Sigma Aldrich, St. Louis, MO, USA) and cultured for an additional 48 h in serum-reduced (3% (*v*/*v*) FCS) endothelial cell media (EBM2, #00190860, Lonza, Basel, Switzerland). The media were centrifuged (1200 rpm, 5 min, room temperature (RT)) to remove the dead cells. The resulting supernatant was used as CM and stored at −20 °C.

### 4.3. Static Cell Adhesion Assay

To analyze the adhesive capacity of tumor cells to brain endothelial cells, static cell adhesion assays were performed. Briefly, tumor cells were cultured to a confluence of 70% and labeled with CellTracker^TM^ Green CMFDA (#C7025, Invitrogen, Waltham, MA, USA) according to the manufacturer´s protocol. A quantity of 2.3 × 10^4^ cells were seeded in black 96-well plates and grown to confluence for 48 h. In the case of TNF-α treatment, hBMECs were treated with 10 ng/mL TNF-α (diluted in endothelial cell basal medium) for 4 h at 37 °C, followed by the seeding of 5 × 10^4^ tumor cells on top and incubation for 40 min at 37 °C. Wells were washed 3 times with PBS containing MgCl_2_ and CaCl_2_ (PBS (+/+)). Fluorescence was measured with a fluorescence plate reader (SynergyH1, BioTek, Winooski, VT, USA).

### 4.4. Electrical Cell-Substrate Impedance Sensing (ECIS)

The hBMECs were plated (1.2 × 10^5^/ well/100 µL) on 0.5% collagen I-coated, 8-well, gold-plated electrode arrays (8W10E+, Applied Biophysics, Troy, NY, USA). Endothelial cell layer integrity was measured every 48 s at multiple frequencies (500–64,000 Hz) using ECIS (Applied Biophysics, Troy, NY, USA). Cells were grown for approximately 24 h until a constant baseline resistance of 1000 ohms was measured. Breast cancer cells (1.2 × 10^5^ tumor cells; ratio 1:1) were added to the endothelial cell layer and resistance was measured for 12 h. All ECIS data were analyzed using ecis.exe software and plotted as the mean of multiple replicates (minimum of three).

### 4.5. Transwell Invasion Assay

A total of 5 × 10^4^ cells were seeded on Attachment Factor-coated 8 µm pore-size transwell inserts for 24-well plates (#353097, BD Falcon, Franklin Lakes, NJ, USA) and cultured until confluence, approximately for 48 h. A total of 1 × 10^4^ CMFDA-labeled tumor cells were allowed to invade through the hBMEC monolayer for 48 h. Cells were washed with PBS, and noninvaded cells on the upper transwell side were removed by using a cotton swab. Cells on the lower transwell side were fixed for 20 min at room temperature (RT) with 3.7% formaldehyde solution (10% (*v*/*v*) formaldehyde (37%), 50% (*v*/*v*) 0.2 mol Na-phosphate-buffer (pH 7.2–7.4), 40% (*v*/*v*) Aqua dest.) and washed with PBS (+/+). Transwell membranes were taken out and mounted on a glass slide with a DAPI mounting medium (#H-1200, Vectashield, Burlingame, CA, USA). Slides were dried for 2 h in the dark and stored at −20 °C. The invaded cells were counted using a fluorescence microscope, BZ II viewer/analyzer software, and a hybrid cell count tool. For each experiment, the analysis was run in triplicate.

### 4.6. Immunofluorescence

A total of 5 × 10^4^ cells were seeded on sterile coverslips into 24-well plates and cultured for 72 h. Cells were washed with PBS containing MgCl_2_ and CaCl_2_ (PBS (+/+)) and fixed for 20 min at RT with a 3.7% formaldehyde solution. After three washing steps and blocking for 1 h at RT with 1% BSA (*v*/*v*)/ PBS (+/+), cells were stained with an anti-ZO-1 antibody for 4 h at RT (monoclonal rabbit IgG, diluted 1:150 in incubation buffer (0.3% Tween-20, 0.1% BSA in HEPES); #8193, Cell Signaling Technology, Danvers, MA, USA). Washing steps (3 × 5 min with PBS (+/+)) were followed by incubation with a secondary antibody (polyclonal goat anti-rabbit FITC, 1:250 in incubation buffer (0.3% Tween-20, 0.1% BSA in HEPES)) for 1 h at RT in the dark. Nuclei were stained with DAPI (#9542, Sigma Aldrich, St. Louis, MO, USA, 1:1000 in PBS (+/+), 10 min, RT, dark). Coverslips were transferred to glass slides and mounted with Fluoromount-G^TM^ (#00-4958-02, Invitrogen, Waltham, MA, USA). Slides were stored at −20 °C in the dark and imaged with a fluorescence microscope (Keyence BZ-900, Osaka, Japan).

### 4.7. RNA Sequencing

Primary human brain endothelial cells were seeded at a density of 1 × 10^4^ cells in a 6-well plate and grown for 72 h. After treatment with CM of breast cancer cell lines for 4 h, the total RNA was extracted with an RNeasy Mini Kit (Qiagen, Valencia, CA, USA) according to the manufacturer’s protocol. Isolated RNA was dissolved in RNase-free water and stored at −80 °C. The quality of total RNA was evaluated with Agilent 2100 Bioanalyzer and the RNA 6000 Nano Kit (Agilent Technologies Inc., Santa Clara, CA, USA). The RNA concentration was determined with a DS-11 FX Spectrophotometer (DeNovix, Wilmington, DE, USA). MDA-MB-231 and MDA-MB-231-BR were grown to 70–80% confluency in a 6-well plate, followed by RNA isolation, RNA quality control (sample purity for human samples: RNA 28S/18S ≥ 1.0; RIN ≥ 7.0), and RNA concentration measurement as described above. RNA sequencing was performed by BGI Genomics (Shenzhen, China) using the DNBSEQ^TM^ Technology platform. All samples were measured in quadruplets (*n* = 4) and between 20 and 25.8 M paired-end sequence reads of length 100 bp were obtained per replicate. Data analysis was carried out at the Bioinformatics Core of the University Medical Center Hamburg-Eppendorf. Sequence reads were processed with fastp (v0.20.1) (GitHub, Inc., San Francisco, CA, USA) to remove sequences of sequencing adapters, as well as low quality (Phred quality score below 10) sequences from the 3′-end of the sequence reads [77]. Subsequently, reads were aligned to the human (GRCh38.104) or mouse (GRCm39.104) reference assemblies using STAR (v2.7.9a) (GitHub, Inc., San Francisco, CA, USA) [78]. Differential gene expression was assessed with DESeq2 (GitHub, Inc., San Francisco, CA, USA) [79]. A gene was considered to be significantly differentially expressed if the corresponding absolute log2-transformed fold change (log2FC) was not less than 1 and, in addition, the p-value did not exceed a value of 0.05. The detection of pathways overrepresented in the set of differentially expressed genes was performed using clusterProfiler (v4.05) (GitHub, Inc., San Francisco, CA, USA) [80] in combination with the Reactome Pathway [81], KEGG pathways [82], and Gene Ontology databases [83]. Sequence data reported in this publication were submitted to the European Nucleotide Archive (ENA). They are publicly available under accession PRJEB40510.

### 4.8. Flow Cytometry

For flow cytometry analysis 2 × 10^5^ cells were stained for E-selectin and VCAM1 in FACS tubes. A detailed protocol can be found in the Supplementary Materials and Methods.

### 4.9. Quantitative Real-Time PCR

RNA was extracted with RNeasy Mini Kit (Qiagen, Valencia, CA, USA), reverse-transcribed with qScriber^TM^ cDNA Synthesis Kit (#RTK0104, HighQu, Santa Clara, CA, USA) according to the manufacturer’s protocol, and further analyzed by real-time qPCR (StepOnePlus, Applied Biosystems, Foster City, CA, USA). For qPCR with ORATM qPCR Green ROX H Mix (#QPD0201, highQu, Santa Clara, CA, USA) the following primers were used: *VCAM-1* (172-FP TGTTTGCAGCTTCTCAAGCTTTTA; 334-RP GTCACCTTCCCATTCAGTGGA), *SELE* (646-FP CAAGTGTGACCCTGGCTTCA; 795-RP CATGCTGCTTGGCAGGTAAC), *TXNIP* (FP CCCTGAAAAGGTGTACGGCA; RP ATTCTCACCTGTTGGCTGGT), *CXCL1* (908-FP TCACAGTGTGTGGTCAACAT; 1037-RP AGCCCCTTTGTTCTAAGCCA), and *GAPDH* (FP GTCAGTGGTGGACCTGACCT; RP TGCTGTAGCCAAATTCGTTG). Fold change in expression was calculated using the ΔΔCT method. Data were normalized to *GAPDH*.

### 4.10. Intracardiac Metastasis Mouse Model

Female 8 to 9-week-old SCID mice (CB17/lcr-Prkdcscid/lcrlcoCrl) were anesthetized under inhalation anesthesia with 2–3% isoflurane (O_2_ 0.5–0.5 L/ min) and 1 × 10^6^ tumor cells (MCF-7, MDA-MB-231, and MDA-MB-231-BR) were injected intracardially into the left ventricle of the heart (*n* = 15 per group). The tumor cells previously tested as negative for mycoplasma and were transduced by lentiviral transfection with luciferase-bearing plasmid, and bioluminescence signals were tested before injection by the addition of the substrate D-luciferin and measurement of the emerging light signal via bioluminescence imaging as previously described [84]. After intracardiac injection, metastasis formation was monitored weekly under bioluminescence imaging (BLI). Therefore, anesthetized mice were imaged from both dorsal and ventral views approximately 10–15 min after intraperitoneal injection of D-luciferin using the IVIS^®^ Spectrum in vivo imaging system (PerkinElmer, Waltham, MA, USA). The success of the intracardiac injection was indicated on day 7 by images showing systemic bioluminescence throughout the animal. Mice showing an insufficient injection were sacrificed. Assessment of subsequent metastasis was monitored in vivo weekly by imaging for up to 3 weeks. Mice showing termination criteria (a weight loss of more than 10% and generally conspicuous symptoms such as stilted walking, separation from the group, and erect, shaggy coat) were immediately sacrificed. At the endpoint, animals were anesthetized with ketamine/xylazine anesthesia (application volume of 10 mL/kg mouse; i.p.) and blood was collected from the left ventricle by cardiac puncture immediately before the final killing was executed by cervical dislocation. Ex vivo bioluminescence imaging was conducted from the heart, lungs, kidneys, ovaries, spleen, brain, liver, and gastrointestinal tract. Bone marrow was isolated from the left femur and tibia, and the whole brain and peripheral organs were paraffin-embedded for further analysis as previously described [85]. The animal experiments were approved by the Authority for Social Affairs, Family, Health, and Consumer Protection of the Free and Hanseatic City of Hamburg through application N005/2020.

### 4.11. Embedding of Murine Brains in Paraffin and Luciferase Histochemistry

To confirm the brain metastatic lesions, a luciferase assay was performed. Therefore, the murine brain tissue was fixed overnight in 3.7% phosphate-buffered formalin and then dehydrated in an automated tissue processor. After dehydration, the tissue was incubated in liquid paraffin in a pouring device twice for 5 h at 63 °C. The paraffin-impregnated tissue pieces were transferred to metal molds with paraffin at 63 °C and cooled down. The FFPE (Formalin-fixed, paraffin-embedded) tissue sections with a thickness of 4 µm were prepared using a microtome and mounted on glass slides. The sections were fixed to the glass slides overnight in a heating oven at 37 °C. IHC was performed as previously described [86]. Briefly, antigen retrieval was performed using 1× citrate buffer (pH 6) in a steamer (100 °C, 20 min), and subsequent incubation with luciferase primary antibody (ab181640, Abcam, Cambridge, UK) was performed (1:500 in antibody diluent (Dako, Agilent Technologies Inc., Santa Clara, CA, USA), overnight at 4 °C). An incubation step with biotinylated anti-Goat IgG secondary antibody (BA-9500, Vector Laboratories, Burlingame, CA, USA; 10 ml TBS, 50 µL normal horse serum and 100 µL anti-goat IgG) for 30 min at RT was followed by luciferase-protein visualization using the ABC-AP Kit (30 min.) and the permanent AP Red Kit. Counterstaining of the nuclei was performed by incubation in Mayer’s hematoxylin for about 5 s followed by bluing in tap water.

### 4.12. Statistics

Statistical analysis for all in vitro and in vivo experiments, except the RNA sequencing results, was conducted using SPSS Version 25 (IBM SPSS Statistics, Armonk, NY, USA). For all functional assays, cells were plated in triplicate and each experiment was performed at least three times. Statistical significance was determined using unpaired, two-tailed Student´s t-tests. The assumption of homogeneity of variance was tested using Levene´s test of equality of variances (*p* > 0.05). Results are given as mean ± s.d. All p-values lower than 0.05 were considered statistically significant.

## 5. Conclusions

In summary, we observed a significantly higher ability of triple-negative BC (TNBC) cells (MDA-MB-231) and the brain-seeking cell subline (MDA-MB-231-BR) to adhere to brain endothelial cells (hBMEC), to disrupt the BBB integrity, and to invade, compared to luminal BC cells (MCF-7). Further, the brain-seeking subline and parental TNBC cells behaved similarly in terms of adhesion, whereas the first showed a stronger impact on the brain endothelium integrity and increased invasive ability. An in vivo model using an intracardiac injection in SCID mice corroborated these findings. The impact of the three BC cell lines on the BBB was precisely deciphered at the molecular level by transcriptome analysis of hBMECs after cocultivation with BC cell secretomes. Thus, an entirely different regulation of endothelial genes was observed, including upregulation of adhesion molecules (*SELE* and *VCAM1*), chemokines (*CXCL1/2/3/8*), and downregulation of gap junction proteins (*GJA4* and *GJA5*). Four pathways were specifically deregulated in the brain endothelial cells by the brain-seeking cell line in comparison with untreated ECs, namely signaling by NTRK1, interleukin-7 signaling, interleukin-6 family signaling, and gap junction assembly. However, most factors included in these pathways were significantly deregulated by both TNBC cell lines, but to a higher extent by the brain-seeking cell line. Two factors related to angiogenesis activation, namely *TNXIP* and *CXCL1*, were identified to be significantly deregulated in hBMECs by the brain-seeking subline in comparison with the parental TNBC cells and, therefore, considered as brain-metastasis specific. The transcriptomes of the two aforementioned TNBC cell lines strongly differed as well. Thus, the deregulation of certain angiogenesis-associated factors (*PTGS2*, *SLIT2*, *JAG1*, *RECK*, and *BMPER*) and several genes related to cell migration and invasion (*PTGS2*, *JAG1*, *PDGFD*, *MISP*, *HES1*, *NRG1, ITGB3*, *DOCK10*, *GPC6*, *FSCN1*, *DACH1, RHOU, RECK*, and *DLC1*) might explain the more aggressive phenotype observed for the brain-seeking cell line in vitro and in vivo.

## Data Availability

Sequence data reported in this publication were submitted to the European Nucleotide Archive (ENA). They are publicly available under accession PRJEB40510.

## Supplementary Materials

The following supporting information can be downloaded at: https://www.mdpi.com/article/10.3390/ijms23031900/s1.
