# Supplementary material for: Insights into the Steps of Breast Cancer–Brain Metastases Development: Tumor Cell Interactions with the Blood–Brain Barrier
Source: Int J Mol Sci. 2022 Feb 8;23(3):1900. doi: 10.3390/ijms23031900 (PMC8836543; doi:10.3390/ijms23031900)
Supplement: Supplementary file 1 [file ijms-23-01900-s001.zip › Supplementary materials & methods.pdf]

Flow cytometry

For flow cytometry analysis $2x10^5$ cells were stained for E-selectin and VCAM1 in FACS tubes. Cells were washed once with PBS (+/+) (centrifuged for 5 min, 1200 rpm, 4°C) and stained with fluorochrome- conjugated antibody for 30 min at 4°C in the dark. After a washing step (PBS (+/+), centrifuged) cells were suspended in 500 µl 1 % BSA (v/v)/ PBS (+/+), and measured with FACSCalibur (Becton Dickinson). The analysis was performed with FlowJoV10 Software.

- E-Selectin (E-Selectin-PE, Mouse Anti-Human CD62E, Clone: 68-5H11, BD Biosciences; diluted 1:100 with 1 % BSA (v/v)/PBS (+/+)
- VCAM1 (VCAM1-PE, #12-1069-41, ThermoFisher Scientific; diluted 1:25 with 1 % BSA (v/v)/PBS (+/+)
