# Supplementary figures and images for: Insights into the Steps of Breast Cancer–Brain Metastases Development: Tumor Cell Interactions with the Blood–Brain Barrier
Source: Int J Mol Sci. 2022 Feb 8;23(3):1900. doi: 10.3390/ijms23031900 (PMC8836543; doi:10.3390/ijms23031900)

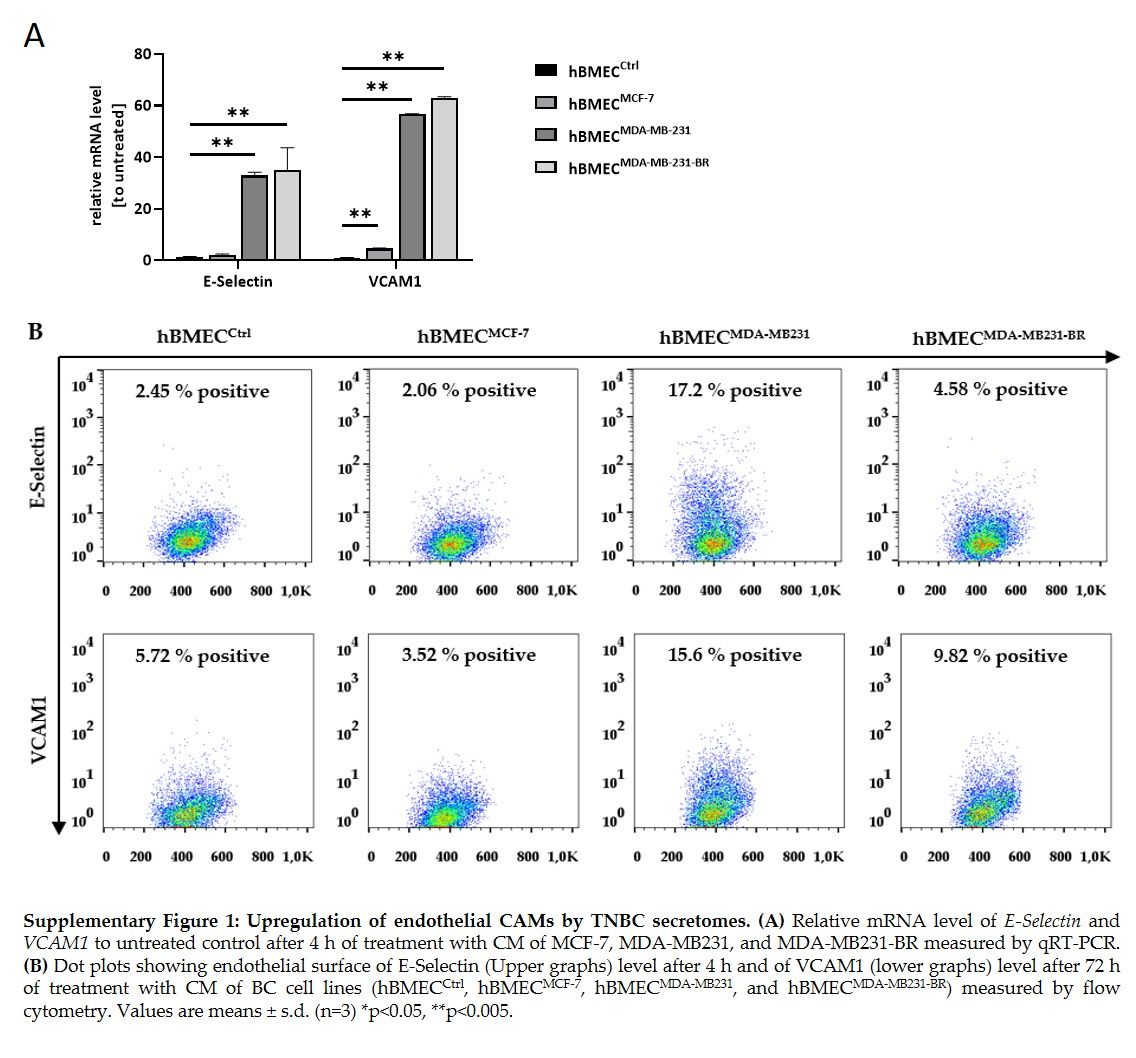

Supplement: Supplementary file 1 [file ijms-23-01900-s001.zip › Figure S1.jpg]
